# Feasibility of Real-Time Near-Infrared Fluorescence Tracer Imaging in Sentinel Node Biopsy for Oral Cavity Cancer Patients

**DOI:** 10.1245/s10434-015-4883-7

**Published:** 2015-10-14

**Authors:** Anders Christensen, Karina Juhl, Birgitte Charabi, Jann Mortensen, Katalin Kiss, Andreas Kjær, Christian von Buchwald

**Affiliations:** Department of Otolaryngology, Head & Neck Surgery and Audiology, Rigshospitalet, Copenhagen University Hospital, Copenhagen, Denmark; Department of Clinical Physiology, Nuclear Medicine & PET and Cluster for Molecular Imaging, Rigshospitalet and University of Copenhagen, Copenhagen, Denmark; Department of Pathology, Rigshospitalet, Copenhagen University Hospital, Copenhagen, Denmark

## Abstract

**Background:**

Sentinel node biopsy (SNB) is an established method in oral squamous cell carcinoma (OSCC) for staging the cN0 neck and to select patients who will benefit from a neck dissection. Near-infrared fluorescence (NIRF) imaging has the potential to improve the SNB procedure by facilitating intraoperative visual identification of the sentinel lymph node (SN). The purpose of this study was to evaluate the feasibility of fluorescence tracer imaging for SN detection in conjunction with conventional radio-guided technique.

**Methods:**

Prospective study of patients with primary OSCC planned for tumor resection and SNB. Thirty patients were injected peritumorally with a bimodal tracer (ICG-^99m^Tc-Nanocoll) followed by lymphoscintigraphy and SPECT/CT to define the SNs and their anatomical location preoperatively. SNs were detected intraoperatively with a hand-held gamma-probe and a hand-held NIRF camera.

**Results:**

In 29 of 30 subjects (97%), all preoperatively defined SNs could be identified intraoperatively using a combination of radioactive and fluorescence guidance. A total of 94 SNs (mean 3, range 1–5) that were both radioactive and fluorescent ex vivo were harvested. Eleven of 94 SNs (12%) could only be identified in vivo using NIRF imaging, and the majority of those were located in level 1 close to the primary tumor.

**Conclusions:**

A combined fluorescent and radioactive tracer for SNB is feasible, and the additional use of NIRF imaging may improve the accuracy of SN identification in oral cancer patients. Intraoperative fluorescence guidance seems of particular value when SNs are located in close proximity to the injection site.

In head and neck cancer, the presence of metastasis in the tumor draining lymph nodes in the neck is one of the most important prognostic factors for treatment outcome.^1^,^2^ Sentinel node biopsy (SNB) is gaining acceptance in oral squamous cell carcinoma (OSCC) and has been applied as a standard staging method for the cN0 neck since 2007 in our center.^3^ Approximately 20–30 % of patients presenting with a cN0 neck harbor subclinical (occult) lymph node metastases and can be identified using SNB, whereas the remaining majority of patients can be spared an unnecessary selective neck dissection and the associated morbidities.^4^,^5^ A recent meta-analysis of SNB for early OSCC comprising 987 patients showed a sensitivity and negative predictive value of 86 and 94 %, respectively.^6^ The accuracy of conventional radiocolloid-based SNB depends heavily on the ability to correctly identify the true SN intraoperatively guided by a radioactive signal combined with information from preoperative lymphoscintigraphy imaging. Radioactivity is characterized by high tissue penetration but limited spatial resolution, which may affect the accuracy of the intraoperative navigation towards the SN. In particular when the SN is located in close proximity to the injection site where background radioactivity is high, navigation can be difficult or even impossible. In the setting of OSCC, this problem is especially the case for SNs located in level 1 due to complex drainage and close spatial relation to the oral cavity.^7^ A lower accuracy of the SNB procedure for tumors located in the floor of the mouth (FOM) has been reported in recent larger series, which may be explained by masking of the SN due to radioactive shine-through from the injection site.^8^–^10^ Near-infrared fluorescence (NIRF)-guided SNB is a novel modality that enables real time intraoperative imaging of the lymphatics within the surgical field with high spatial resolution.^11^,^12^

The utility of Indocyanine Green (ICG) for SN mapping has been studied in multiple malignancies suited for the SNB procedure and most comprehensively in breast cancer.^13^–^15^ Recently, a bimodal approach has been introduced for SNB by exploiting the beneficial hydrodynamic draining properties of radiocolloids and the high affinity of ICG for albumin, thereby assembling a combined tracer complex, which is both fluorescent and radioactive.^16^ With a bimodal tracer the preoperative radioactivity-based imaging and surgical planning can be directly combined with the intraoperative detection of the SN with both a visible fluorescent signal and the conventional radioactive (acoustic) signal available for navigation.^17^ Accordingly, adding NIRF imaging to the SNB procedure has the potential to improve the accuracy of intraoperative SN detection in OSCC.

The purpose of this study was therefore to evaluate the feasibility of NIRF imaging for SN detection in early oral cancer in conjunction with conventional radioguided technique.

## Patients and Methods

Between December 2013 and September 2014, 30 patients diagnosed consecutively with primary cN0 T1-T2 OSCC and scheduled for tumor resection and SNB were included in the study. In all the patients, the nodal neck status was evaluated with clinical examination, ultrasound, CT, and/or MRI. Exclusion criteria were prior head and neck cancer, prior surgery or radiotherapy to neck, pregnancy, severe kidney failure, and allergy to ICG or iodine. Patients were enrolled after obtaining informed consent. The first author (AC) assisted in all the operations. The study protocol was conducted in accordance with the Helsinki Declaration and approved by the Danish Regional Scientific Ethical Committee (H-1-2013-091). The study was approved as a single-institution clinical trial by the European Medicines Agency (EudraCT No. 2013-003578-28, www.clinicaltrialsregister.eu) and was monitored and conducted in accordance with GCP standards.

### Tracer Preparation and Administration

^99m^Tc-Nanocoll was prepared by adding 2 GBq of pertechnetate in 2 ml of saline to a vial of Nanocoll containing 0.5 mg of human albumin colloid (GE Healthcare). The mixture was incubated for 30 min before use. ICG was prepared by adding 5 ml of sterile water to a vial containing 25 mg of dry compound (ICG-Pulsion, Pulsion Medical, Germany) and further diluted to a 0.5 mg/ml of ICG stock solution. Then, 0.1 ml of ^99m^Tc-Nanocoll (55 or 110 MBq depending on same-day or day-before surgery) and 0.1 ml of ICG stock solution was mixed to a final volume of 0.2 ml of ICG-^99m^Tc-Nanocoll containing 0.05 mg of ICG (0.32 mM). The tracer was injected submucosally in four peritumoral deposits.

### Preoperative Imaging

Lymphoscintigraphy (LSG) and SPECT/CT were performed using a dual head SPECT/CT camera (Precedence SPECT/CT, Philips Healthcare, The Netherlands) in a 60-min scanning protocol. Immediately after tracer injection, a dynamic LSG in anterior and lateral projection was acquired followed by a static LSG 15 min postinjection to identify the temporal order of SN identification. Then, SPECT/CT was performed and images were generated. If the first scanning session did not identify any SN, a second late LSG and SPECT/CT was performed 120 min postinjection. Images were interpreted by a specialist in nuclear medicine, and the localization of the SN was depicted in an anatomical chart of the neck divided into neck levels as proposed by AHNS.^18^ The number of SNs detected on LSG and SPECT/CT was recorded. A lymph node clearly visible on LSG or SPECT/CT was considered a SN.

### Surgical Procedure and Intraoperative Imaging

Tumor resection and reconstruction was done before the SNB neck procedure. Fluorescence imaging was performed with the clinically approved hand-held NIR camera system and accompanying software (Fluobeam 800, Fluoptics, France). Before skin incision, fluorescence imaging was performed to localize SNs that were visible transcutaneously. During surgery, the NIR camera and a hand-held gamma probe (Neo2000, Neoprope Corporation, USA) were used in parallel to navigate towards the SN, and the modality (radioactive and/or fluorescent) for identification was recorded. To distinguish a SN from a non-SN, a radioactive count rate less than 10 % of the SN with the highest count rate was applied as a rule. In addition, any lymph node with a bright fluorescent signal was appointed a SN. Intraoperative fluorescence imaging of the SNs in the in situ location was performed before removal, and the exact anatomical location was recorded. The surgical field was searched systematically for any remaining radioactive or fluorescent signal before closure. Operation time for the SNB in the neck was defined as from skin incision to completed skin closure.

### Ex Vivo Imaging and Histology

The harvested SNs were postoperatively imaged with the NIR camera under standardized conditions and aligned beside a reference tube containing a 0.2-µM ICG solution. Harvested SNs were fixed in formalin for 24 h, paraffin embedded, and processed with step-serial sectioning, using H&E staining and cytokeratin antibodies according to a previously described protocol.^4^ Tumor deposits were classified as macrometastasis, micrometastasis, or isolated tumor cells.^19^ Non-SNs encountered during dissection that not could be preserved and therefore resected were examined with routine histopathological analysis. Tissue sections stained with H&E from a selected number of SNs and non-SNs were imaged using a NIR scanner (Odyssey Sa, LI-COR Biosciences, UK).

### Data Analysis

For statistical analyses, SAS Enterprise software Version 9.4 was used. A value of *p* < 0.05 was considered statistically significant. Continuous measures were compared using Wilcoxon signed-rank test between groups. On intraoperative and postoperative unsaturated raw grayscale images the fluorescence signal was semiquantified using regions of interest in the software ImageJ (version 1.48, NIH, USA). A Signal-to-Background-Ratio (SBR) and Signal-to-Reference-Ratio (SRR) was calculated by dividing the fluorescent signal of the SN by the fluorescent signal of adjacent tissue intraoperatively or the ICG-reference and standard background postoperatively.

## Results

Patient and tumor characteristics are outlined in Table 1. All patients were managed with a transoral tumor resection and a primary closure or a local flap. No adverse reactions or complications related to the hybrid tracer were observed. Six patients (20 %) had a single positive SN and a subsequent selective neck dissection was performed where no additional metastatic deposits were found in the neck specimens on routine histopathology.

**Table 1:** Patient and tumor characteristics

Characteristic	N or mean	% or range
Age, year (mean, range)		
Men	64	(52–83)
Women	63	(50–79)
Body mass index (mean, range)	24.3	(18.0–32.3)
Tumor subsite location in oral cavity		
Floor of mouth	15	50
Tongue, ant. 2/3	9	30
Inferior tongue	2	7
Buccal	1	3
Hard palate	1	3
Gingiva	1	3
Retromolar trigone	1	3
Tumor crossing midline		
Yes	8	27
No	22	73
Tumor stage		
T1	18	60
T2	12	40
Operation time (min) for SNB neck procedure (mean, range)	39	(15–115)
Pathology		
−SN	88	94
+SN	6	6
Macrometastasis (>2 mm)	3	50
Micrometastasis (≥0.2 mm, ≤2 mm)	3	50
Isolated tumor cells (<0.2 mm)	0	0
pN staging		
pN0	24	80
pN1	6	20

### Preoperative Imaging

A total of 68 SNs were defined preoperatively and modality for detection is shown in Table 2. In two patients, no SN was detected on the initial imaging and a late second LSG and SPECT/CT was performed where a SN could be detected in both cases. In 18 patients (60 %), bilateral lymphatic drainage was seen, and 1 patient (3 %) with a lateralized FOM tumor had only contralateral drainage.

**Table 2:** Modality for SN identification

Modality for SN identification	*N*	Detection rate % (*n*/*n*)
Preoperatively		
Total no. of SNs visualized	68	71 (68/96)
LSG	41	43 (41/96)
SPECT/CT	68	71 (68/96)
Intraoperatively		
Total no. of SNs harvested	94	98 (94/96)
Fluorescent + radioactive	83	86 (83/96)
Radioactivity only	0	0 (0/96)
Fluorescence only	11	11 (11/96)

### Intraoperative Imaging and Lymph Node Detection

**Fig. 1:**
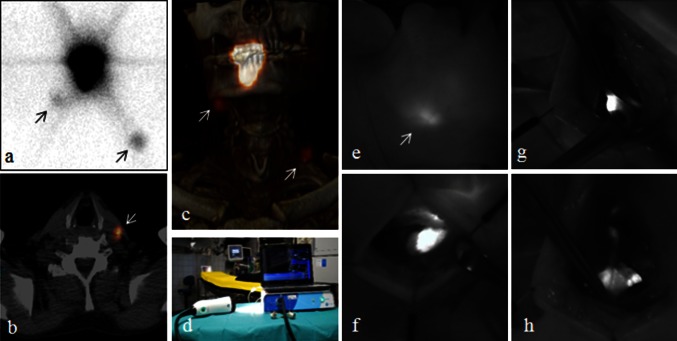
Preoperative and intraoperative SN imaging. LSG (**a**), axial SPECT/CT (**b**), and the 3-D reconstructed SPECT/CT (**c**) in a patient with a well-lateralized tumor on the right anterior tongue showing tracer drainage to an ipsilateral SN in level 1 and directly to a contralateral SN in level 3 (*arrows*). The latter SN in level 3 in the left neck side was visible transcutaneously (**e**). The Fluobeam 800 NIR camera (**d**) designed for ICG imaging. The NIR camera entered the surgical field in a sterile cover, and real-time video imaging was presented for the surgical team on a clinical screen. When using the NIR camera intraoperatively, the direct surgical light was turned off to improve the quality of the imaging. The system has a 750-nm excitation laser and LED white light illumination of the surgical field that does not inflict on the NIRF imaging. The hand-held camera head is maneuverable in all angles and has a ×10 zoom function. The autofocus function allows for flexible working distance. Intraoperative NIRF-guided identification and resection of SN (**f**, **g**, **h**)

A total of 94 SNs were detected intraoperatively yielding an average of 3 SNs (range 1–5) per patient; the distribution in the neck is depicted in Table 3. At least one SN was identified in all patients. In one patient with a FOM tumor, two of three preoperatively defined SNs, with an unusual location medial to the mandible within the FOM, could not be located, and the neck procedure was extended to a bilateral neck dissection after which the patient was staged as pN0. Therefore, the intraoperative detection rate of the preoperatively defined SNs was 97 % (66/68). Before skin incision, 10 % (9/94) of the excised SNs could be visualized by fluorescence transcutaneously (Fig. 1). The success of noninvasive transcutaneous SN identification was significantly correlated with a low BMI (*p* = 0.05).

**Table 3:** Distribution of all harvested SNs stratified by neck side and tumor subsite

Subsite	Ipsilateral neck side by level					Contralateral neck side by level				
	1	2	3	4	5	1	2	3	4	5
FOM	14% (7/50)	42% (21/50)	16% (8/50)	4% (2/50)	2% (1/50)	10% (5/50)	8% (4/50)	4% (2/50)	–	–
Tongue*	6% (2/34)	50% (17/34)	32% (11/34)	3% (1/34)	–	3% (1/34)	–	6% (2/34)	–	–
Other subsites**	50% (5/10)	30% (3/10)	10% (1/10)	–	–	–	10% (1/10)	–	–	–
All subsites	15% (14/94)	43% (40/94)	21% (20/94)	3% (3/94)	1% (1/94)	6% (6/94)	5% (5/94)	4% (4/94)	–	–

FOM tumors showed a higher frequency of lymphatic drainage to both the contralateral level 1 and 2 compared with tongue tumors

*FOM* floor of mouth

* Anterior 2/3 of tongue and inferior tongue

** Buccal, hard palate, gingiva and retromolar trigone

**Fig. 2:**
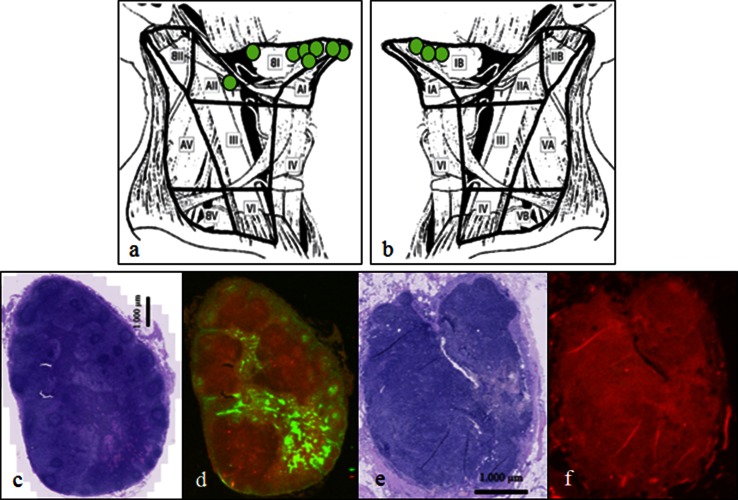
Intraoperative SN identification by NIRF imaging only. The exact anatomical location and neck level location in the ipsilateral (**a**) and contralateral (**b**) neck side of the 11 additional SNs identified only by fluorescence. H&E staining and fluorescence microimaging of a tissue section from a SN (**c** and **d**) and a non-SN (**e** and **f**) located intimately within the same cluster of lymph nodes in level 2a. None of the lymph nodes contains metastatic tumor. In the SN the microantomical distribution of the fluorescent tracer draining from the marginal sinus towards the medullary sinus is visualized. The non-SN is without any signal from ICG on NIRF microimaging

Intraoperatively, 11 SNs in 9 patients could only be identified due to fluorescence. They were all located solitary and not as a part of a cluster of lymph nodes and they were not identified on SPECT/CT (Fig. 2). In seven of nine of the patients, these additional nodes could be detected through the incision made to search level 1 and/or level 2. In two patients with FOM tumors, an additional incision was made in the contralateral neck because transcutaneous NIR imaging demonstrated drainage to level 1, and two additional nodes could be retrieved. These additional SNs were all negative on histology and therefore did not lead to upstaging of patients. No SN was identified only due to the radioactive signal. Ex vivo, all 94 harvested SNs were both radioactive and fluorescent (Table 3). The mean intraoperative SBR of the SNs was 7.8 (range 1.9–30.6). Clusters of 3–5 lymph nodes were often encountered in level 2a where fluorescence enabled easy separation in SNs and non-SNs due to real-time imaging. On micro-fluorescence scanning of tissue slides of SNs and non-SNs anatomically located closely adjacent in a cluster, the fluorescent signal from ICG could be seen distributed within the lymphatic drainage system in the SN while no fluorescent signal could be detected in the non-SN (Fig. 2).

**Fig. 3:**
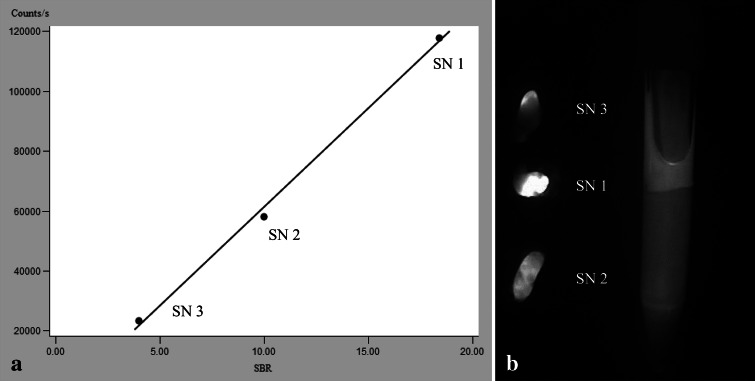
Correlation between the fluorescent and the radioactive signal. A representative case example (**a**) of linear correlation between the intraoperative fluorescent and the radioactive signal in three resected SNs from the same patient. Postoperative NIRF imaging (**b**) of the same three SNs aligned besides a reference tube containing a 0.2-μM ICG concentration

In the 23 patients, who had three or more SNs removed, the fluorescence intensity, measured as SBR or SRR, correlated with the radioactive count both intraoperatively (*p* = 0.015) and ex vivo (*p* = 0.007) when analyzed in a mixed model. Within each patient, a linear correlation between either intraoperative or ex vivo fluorescence intensity and radioactivity was observed (Fig. 3).

## Discussion

To our knowledge, this prospective study is the largest to date on fluorescence-guided SNB in OSCC. By adding the fluorescence modality to the SNB procedure, an additional 11 SNs were detected that otherwise would have been left behind when using conventional radioguided technique. Anatomically, the additional SNs were located almost exclusively in level 1 close to the injection site in the oral cavity and, therefore, demonstrate that intraoperative fluorescence imaging enables navigation towards SNs otherwise masked by the radioactive background signal. These findings support a recent similar study of SN detection in 14 N0 OSCC patients using ICG-^99m^Tc-Nanocoll.^20^ In addition, an added value of the SNB hybrid tracer principle for melanoma in the head and neck region has been reported.^21^

We found SPECT/CT superior to LSG in terms of preoperative SN detection, which previously has been reported by our group and others.^22^,^23^ In addition, SPECT/CT provided very accurate anatomical information about SN location useful for planning of surgery, and we recommend SPECT/CT to be an integrated part of SNB procedures in the head and neck region.

The yield of subclinical metastases was 20 %, which is low compared with other series that reflect that 60 % were T1 tumors with a lower risk of metastatic spread compared with higher T-stage tumors. We observed a high percentage (60 %) of bilateral drainage probably, because the majority of the tumors in this cohort was in the FOM.

We observed an excellent retention of ICG-^99m^Tc-Nanocoll in the SN, even in patients injected the day before the SNB procedure, while draining to second tier nodes downstream was minimal. This probably reflects that the hybrid tracer has the beneficial hydrodynamic characteristics of Nanocoll for SN mapping.^24^ In addition, the strong correlation between fluorescence intensity and radioactivity in the SNs within each patient in our study indicates that the hybrid tracer complex remains stable in vivo. This is in accordance with results reported by van der Poel et al. in prostate cancer patients.^25^ For future studies, this observation also has the implication that only nodes that are both fluorescent and radioactive after resection should be regarded as true SNs.

NIRF-guided SNB mapping using unmixed ICG in oral and oropharyngeal cancer has been reported in a few studies where problems with rapid transportation of ICG to higher echelon nodes were noted.^26^,^27^ When performing NIRF-guided SNB in head and neck cancer patients, strong tracer retention in the SN and minimal overflow to higher echelon nodes is of paramount importance, which depends on the use of a carrier colloid. In breast cancer, the use of unmixed ICG, which has a hydrodynamic diameter <1 nm, has proven to be successful, because direct transcutaneous dynamic visualization of the rapid tracer flow towards the SN within minutes is possible.^28^ However, this is not the case in the neck, and in our opinion initial radioguidance is still required for lymphatic SN mapping in head and neck cancer.

Similar to results by Crane et al. using unmixed ICG for SNB in vulvar cancer, we found the transcutaneous fluorescence signal to be correlated to the BMI of the patient and that transcutaneous node mapping was not feasible.^29^ The fluorescent signal from ICG has a limited tissue penetration of approximately 0.5–1.5 cm. Accordingly, in our study some exploration in the surgical field was necessary in the majority of cases to obtain successful NIRF imaging of the SN.

Clusters of closely located lymph nodes embedded in fatty tissue in the neck are common, and in the present study NIRF imaging enabled direct visual differentiation of SN from non-SNs for selective dissection of the SN. In addition, often only one of several lymph nodes within a cluster was the tumor draining SN. This supports the SNB theory of timely ordered and individual lymph node drainage patterns. By the NIRF imaging approach, the risk of sending non-SNs for expensive thorough SN pathology examination may be limited.

To prove an added value of NIRF-guided SNB in head and neck cancer, future studies with follow-up data of N-site recurrences in SN-negative patients are needed to address the false-negative rate stratified for tumor subsites.

In conclusion, a combined fluorescent and radioactive tracer approach for SNB is feasible and additional use of NIRF imaging may improve the accuracy of SN identification in OSCC. Intraoperative fluorescence guidance seems of particular value when SNs are located in close proximity to the injection site.
